# Tracing the temporal stability of autism spectrum diagnosis and severity as measured by the Autism Diagnostic Observation Schedule: A systematic review and meta-analysis

**DOI:** 10.1371/journal.pone.0183160

**Published:** 2017-09-21

**Authors:** Łucja Bieleninik, Maj-Britt Posserud, Monika Geretsegger, Grace Thompson, Cochavit Elefant, Christian Gold

**Affiliations:** 1 GAMUT–The Grieg Academy Music Therapy Research Centre, Uni Research Health, Bergen, Norway; 2 Department of Child and Adolescent Psychiatry, Division of Psychiatry, Haukeland University Hospital, Bergen, Norway; 3 Department of Clinical Medicine, Faculty of Medicine and Dentistry, University of Bergen, Bergen, Norway; 4 Melbourne Conservatorium of Music, the University of Melbourne, Melbourne, Australia; 5 School for Creative Arts Therapies, University of Haifa, Haifa, Israel; Universite de Bretagne Occidentale, FRANCE

## Abstract

**Background:**

Exploring ways to improve the trajectory and symptoms of autism spectrum disorder is prevalent in research, but less is known about the natural prognosis of autism spectrum disorder and course of symptoms. The objective of this study was to examine the temporal stability of autism spectrum disorder and autism diagnosis, and the longitudinal trajectories of autism core symptom severity. We furthermore sought to identify possible predictors for change.

**Methods:**

We searched PubMed, PsycInfo, EMBASE, Web of Science, Cochrane Library up to October 2015 for prospective cohort studies addressing the autism spectrum disorder/autism diagnostic stability, and prospective studies of intervention effects. We included people of all ages with autism spectrum disorder/autism or at risk of having autism spectrum disorder, who were diagnosed and followed up for at least 12 months using the Autism Diagnostic Observation Schedule (ADOS). Both continuous ADOS scores and dichotomous diagnostic categories were pooled in random-effects meta-analysis and meta-regression.

**Results:**

Of 1443 abstracts screened, 44 were eligible of which 40 studies contained appropriate data for meta-analysis. A total of 5771 participants from 7 months of age to 16.5 years were included. Our analyses showed no change in ADOS scores across time as measured by Calibrated Severity Scores (mean difference [MD] = 0.05, 95% CI -0.26 to 0.36). We observed a minor but statistically significant change in ADOS total raw scores (MD = -1.51, 95% CI -2.70 to -0.32). There was no improvement in restricted and repetitive behaviours (standardised MD [SMD] = -0.04, 95% CI -0.19 to 0.11), but a minor improvement in social affect over time (SMD = -0.31, 95% CI -0.50 to -0.12). No changes were observed for meeting the autism spectrum disorder criteria over time (risk difference [RD] = -0.01, 95% CI -0.03 to 0.01), but a significant change for meeting autism criteria over time (RD = -0.18, 95% CI -0.29 to -0.07). On average, there was a high heterogeneity between studies (I^2^ range: 65.3% to 93.1%).

**Discussion:**

While 18% of participants shifted from autism to autism spectrum disorder diagnosis, the overall autism spectrum disorder prevalence was unchanged.

Overall autism core symptoms were remarkably stable over time across childhood indicating that intervention studies should focus on other areas, such as quality of life and adaptive functioning. However, due to high heterogeneity between studies and a number of limitations in the studies, the results need to be interpreted with caution.

## Introduction

Autism spectrum disorder is a neurodevelopmental disorder defined by persistent social and communication deficits and symptoms with associated restricted and repetitive behaviours and interests. Autism spectrum disorder is estimated to affect more than 1% of people worldwide, and to carry enormous cost for society and for the individual in loss of productive years and cost of educational support [1,2]. Longitudinal studies indicate that only 1 out of 5 individuals with autism spectrum disorder seem to obtain a good adult outcome as indicated by the quality of independent living, friendships and participation in employment [3]. Early intervention is believed to ameliorate this dire outlook, but the evidence for the long-term advantage of total population screening and intervention is still limited [4]. The lack of knowledge on the natural progression of autism spectrum disorder is one of the many challenges in research. Results of previous systematic reviews have suggested that autism is a stable diagnosis even before three years of age, although some children with cognitive impairments may initially be misclassified [5]. However, tracking autism spectrum disorder symptoms is hampered by the lack of valid and reliable measures of symptoms across the life-span and developmental level. Few measures currently exist to track the temporal stability of autism spectrum disorder and severity of the core symptoms over time, as several commonly used instruments (e.g. the Autism Behaviour Checklist, the Childhood Autism Rating Scale, the Gilliam Autism Rating Scale) are not independent of phenotypic characteristics such as age, IQ, and language level [6]. One exception is the Autism Diagnostic Observation Schedule (ADOS) [7], which has different modules tailored to the language level and age of the individual to ensure consistency of autism severity scores across cognitive levels and different age groups from infants to adults. Together with the Autism Diagnostic Interview-Revised [8], it is the current gold standard of autism spectrum disorder diagnosis worldwide [9]. Based on a semi-structured, clinician-administered assessment of the child’s behaviour, the scoring algorithm broadly classifies individuals into non-autism spectrum disorder, autism spectrum disorder, and autism.

Evaluating treatment effects on a neurodevelopmental disorder in children is intrinsically challenging. As all children develop and mature, it is difficult to reliably ascribe the change to the intervention per se. The ADOS-based Calibrated Severity Scores (CSS) aims to minimize the impact of other factors such as age, language and cognitive ability on the autism severity score. The ADOS CSS has been suggested as the most appropriate measure of outcome for treatment and follow-up studies looking to capture change in symptom severity independent of developmental factors [6]; however ADOS raw totals have been used for many years and are still commonly used. However, little is known about the overall “natural” development of ADOS scores in individuals with autism spectrum disorder, and the magnitude of change that could be expected. To date, there has been no meta-analysis focusing on prospective cohort studies addressing the diagnostic stability over time of autism spectrum disorder/autism as measured by the ADOS. The motivation to undertake this study was thus to examine the temporal stability of autism spectrum disorder/autism diagnostic classification over time as measured by the ADOS and plot longitudinal trajectories of core autism symptom severity using the ADOS. The third aim was to identify possible predictors of change in autism spectrum disorder/autism as classified by the ADOS.

## Methods

### Search strategy

We undertook a comprehensive search following guidelines outlined in the PRISMA Statement (www.prisma-statement.org) (S1 and S2 Checklists). Methods of the analysis and inclusion criteria were specified in advance and documented in a protocol (S1 Protocol). PubMed, PsycInfo, Web of Science, EMBASE, DARE, and the Cochrane Library were searched up to September 10, 2014 for the term “Autism Diagnostic Observation Schedule” by one reviewer (ŁB). An update search was conducted on October 12, 2015. The search was not restricted to any language, reference type, or year of publication. Unpublished studies such as conference abstracts and dissertation abstracts were also included. Reference lists of the included review articles were checked to identify any additional studies.

### Study selection

Titles and abstracts of all references identified were inspected independently by two reviewers (ŁB, MBP) to exclude obviously irrelevant reports. Any disagreement was solved through discussion, and consultation with a third reviewer (CG). The final selection of remaining references was based on full-text assessment by three reviewers independently (ŁB, MG, MBP), with at least two assessing every record. Final inclusion was based on the following inclusion criteria:

1. Participants: individuals of any age (including adults) diagnosed with any autism spectrum disorder as defined in DSM-5 [10], or similarly as based on earlier versions of the DSM or the International Classification of Diseases (ICD). This included childhood autism, atypical autism, Asperger syndrome, and pervasive developmental disorder not otherwise specified (PDD-NOS) and autism spectrum disorder, excluding Rett syndrome. We also included individuals at risk of having autism spectrum disorder (e.g. siblings with autism spectrum disorder or through screening instruments).
2. Baseline assessment: A version of the ADOS (ADOS/ADOS-G/ADOS-2/ADOS toddler version) was used as diagnostic measure.
3. Follow-up assessment: A version of the ADOS was used a second time at least 12 months later. If participants were evaluated using ADOS more than twice, we analysed the longest available time span.
4. Type of study: prospective cohort studies addressing the diagnostic stability over time of autism spectrum disorder/autism; or prospective studies of intervention effects (i.e., randomised, non-randomised controlled, or without a control group).

Duplicate publication detection was based on author names, location and setting, specific details of the interventions, numbers of participants and baseline data; and date and duration of the study. When uncertainties remained, we contacted authors.

### Data collection and extraction process

Data were independently extracted by two authors (GT, CE). They confirmed accuracy using a shared, piloted data extraction sheet on participant characteristics at baseline (diagnosed vs. high risk, number, age at initial diagnosis using ADOS, gender), clinical subgroups, study design, interventions, age at follow up, attrition, and outcome category (ADOS total vs. CSS, ADOS subscales, autism spectrum disorder/autism cut-offs). Any disagreements were resolved by discussion with other reviewers (CG, MG, ŁB) and with study authors when required.

### Quality of included studies

Study quality was rated as low when overall attrition was more than 20%. Randomised controlled trials (RCTs) were rated as “low quality” if there was no blinding. Studies failing to report the total number assessed at baseline and RCT studies failing to report blinding status were labelled as of “uncertain quality”.

### Preparatory data analyses

When study results were split according to clinical characteristics, we prepared them as follows: we retained subgroups assigned prospectively (e.g. to intervention vs. control, or divisions between diagnostic groups) [11–16] because they might contain important information on heterogeneity. For studies that separated participants retrospectively (e.g., into improved or not improved [17] or retrospective diagnostic groups [18,19]), without pooled data available from the paper or the authors, we pooled these subsamples using means and pooled SDs.

### Meta-analysis and meta-regression

Meta-analysis was performed for the following outcomes:

1. Total autism severity: either raw scores using any of the published algorithms, or CSS. We prioritized CSS over raw total scores if both were available for the complete sample.
2. Autism severity, subdomain social affect: social affect subtotal, or social+communication total, or (language and) communication domain & social (interaction) domain, or modified scores thereof.
3. Autism severity, subdomain restricted and repetitive behaviour: restricted and repetitive behaviour subtotal, or modified score thereof.
4. Meeting autism spectrum disorder criteria, based on ADOS cut-off value (i.e. ≥ 4 on the CSS, or ≥ 7 to ≥ 11 in the different modules of ADOS raw totals [6]; differing between earlier and later ADOS versions). (Note that this includes those who also meet the higher cut-off for autism. This is in line with diagnostic criteria, but semantically different from the use in ADOS CSS [6].)
5. Meeting autism criteria, based on ADOS cut-off value (i.e. ≥ 6 on the CSS, or ≥ 9 to ≥ 16 in the different modules of ADOS raw totals [6]; differing between earlier and later ADOS versions).

For continuous variables, we used primarily weighted mean differences (MDs). For ADOS raw scores (which have similar but not identical meaning due to various adaptations, versions, and modules used), we also examined whether using standardized mean differences (SMDs) affected results. This was not necessary for ADOS CSS, where the scores have the same meaning across ADOS modules. The ADOS subscales social affect and restricted and repetitive behaviour were analysed using SMD as they were reported in various forms. Dichotomous variables were evaluated using risk differences (RDs) because they are straightforward to interpret as percentage point change.

We calculated both fixed-effects and random-effects meta-analyses, but report only random effects because heterogeneity between studies was high (I^2^ ≥50%) in all main analyses. As potential predictor variables, we analysed initial age, initial diagnosis (diagnosed vs. high-risk), duration of follow-up, and type of intervention (specific vs. carer training vs. standard care), using random-effects meta-analysis and meta-regression. All analyses were conducted using R version 3.3.1 (http://www.r-project-org) and R package meta.

## Results

### Results of the search

**Fig 1:**
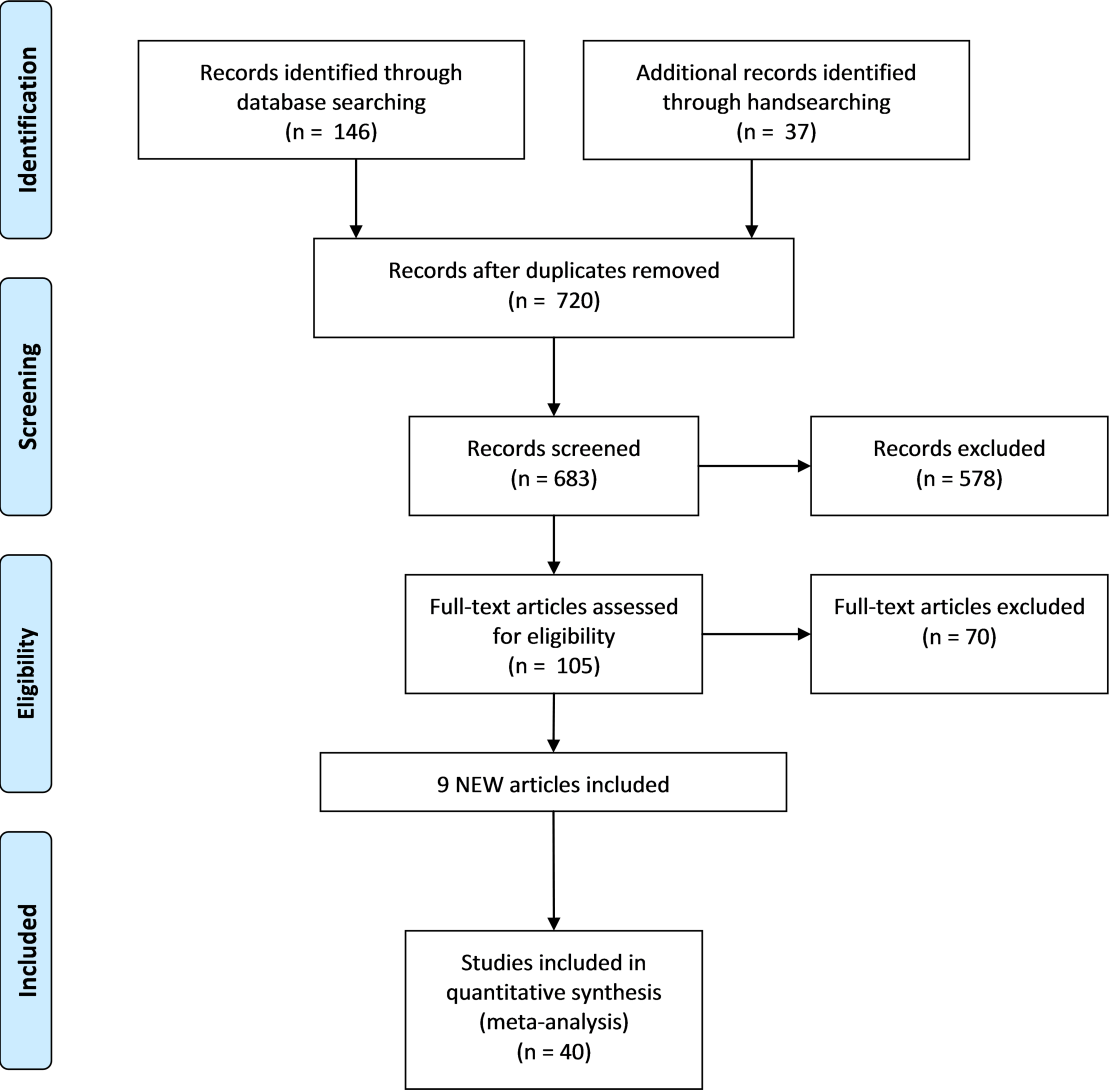
PRISMA flow chart of included studies. Shows the study selection process with numbers excluded at each stage.

The combined literature search yielded 1443 titles. After excluding duplicates and clearly irrelevant papers, we examined full-text articles of 105 potentially relevant studies. The update search yielded 29 new potentially relevant articles of which 9 were included. Finally, 44 articles met inclusion criteria. Four publications referring to already included studies [11,13,18] were merged with those, leaving forty studies appropriate for meta-analysis (Fig 1).

### Characteristics of the included studies

We included 40 studies with a total of 5771 participants (range: 12 to 1241) (Table 1). Age at baseline ranged from seven months [20] to 16.5 years [21], i.e. no studies of adults existed for inclusion. Gender distribution was not always reported, but ranged from 39% [22] to 100% males [23,24]. Ten studies included high-risk children, twenty-nine included children diagnosed with autism spectrum disorder, and one study [15] included separate samples of both types. Some studies also included comparison groups of low-risk children, which were outside the scope of this review. Most studies (n = 35) were prospective cohort studies, whereas five were RCTs. Some study samples were divided either prospectively or retrospectively into clinical subgroups (Table 1). Follow-up duration ranged from 12 months to 17 years [11], with a median of 18.5 months (mean 30.45; SD 34.22).

**Table 1:** Characteristics of included studies.

Study	Participant characteristics at baseline^1^				Design type^2^	Intervention type^3^	Clinical subgroups^4^		Follow-up		Outcomes reported			Data used^5^
	Type^6^	N	Age at baseline in months; mean (range)	Male (%)		(assigned prospectively)	prospective (at baseline)	retrospective (at follow-up)^7^	Duration, months	Attrition (%)	ADOS overall (total or CSS)^8^	ADOS subscales^9^	ASD/AD cut-offs	
Akshoomoff 2004 [23]	Diag.	52	41 (23–58)	100	CS	Standard care	—	—	37	None	—	—	ASD	P
Aldred 2004 [25]	Diag.	28	48 (24–71)	89	RCT	Carer training, standard care	—	—	12	None	Total	SA, RRB	AD	P
Anderson 2014 [11]	Diag.	85	30 (29-NA)	92	CS	Standard care	IQ < 70, IQ ≥ 70	—	204	None	—	SA, RRB	ASD	P
Ben Itzchak 2009 [17]	Diag.	68	25 (18–35)	91	CS	Specific	—	—	12		Total	—	AD	P
Brian 2008 [26]	HR	155	18 (16–20)	47	CS	Standard care	—	—	18		—	—	ASD	P
Chawarska 2009 [18]	Diag.	89	22 (13–27)	NA	CS	Specific		AD, PDD-NOS	26		—	—	AD	P
Chawarska 2014 [19]	HR	719	18 (NA)	57	CS	Standard care	—	—	18		CSS	—	—	P
Dawson 2010 [27]	Diag.	48	24 (18–30)	71	RCT	Specific, standard care	—	—	24	3/48	CSS	—	AD	P
Dereu 2012 [28]	HR	41	30 (25-NA)	63	CS	Specific, standard care	—	True, false positive screens	21	13/41	CSS	—	ASD	P
Di Renzo 2015 [21]	Diag.	90	78 (30–198)	80	CS	Specific	—	—	48		Total	—	ASD, AD	P
Freitag 2012 [29]	Diag.	13	70 (48–92)	77	CS	Specific	—	—	12		CSS	—	—	P
Gammer 2015 [22]	HR	54	24 (NA)	39	CS	Standard care	—	—	12		CSS	—	—	P
Gotham 2012 [30]	Diag.	345	40 (34–90)	82	CS	Standard care	—	—	55		CSS	SA, RRB	ASD, AD	P
Green 2010 [31]	Diag.	152	45 (24–60)	91	RCT	Carer training, standard care	—	—	13		—	SA, RRB	ASD, AD	P
Guthrie 2013 [32]	HR	82	19 (15–24)	78	CS	Standard care	—	—	17		—	SA, RRB	ASD	P
Gutstein 2007 [33]	Diag.	12	61 (21–94)	93	CS	Carer training	—	—	36		—	—	ASD, AD	P
Hobson 2016 [34]	Diag.	18	73 (NA)	89	CS	Carer training	—	—	26		CSS	—	—	P
Howlin 2007 [35]	Diag.	83	82 (47–122)	87	RCT	Carer training, standard care	—	—	16		Total	SA	ASD, AD	P
Kleinman 2008 [12]	Diag.	61	27 (16–35)	86	CS	Standard care	AD, PDD-NOS	—	19		Total	—	—	P
Klintwall 2015 [36]	Diag.	70	22 (NA)	89	CS	Specific	—	—	16		Total	—	—	P
Landa 2013 [13]	Diag.	54	15 (13–16)	81	CS	Standard care	Early, later diag.	—	22	1/54	—	SA, RRB	—	P
Lerna 2014 [24]	Diag.	14	69 (18–60)	100	CS	Specific, standard care	—	—	12		Total	SA	—	P
Lord 2006 [37]	HR	192	24	NA	CS	Standard care	AD, PDD-NOS, TD -		84	20/192	Total (+CSS for subsample)	SA, RRB	AD	U
Lord 2012 [38]	HR	78	17 (12–18)	79	CS	Standard care	—	ASD (severe persistent, worsening, improving), TD	18		Total (+CSS for subsample)	SA, RRB	AD	U
Louwerse 2015 [39]	Diag.	72	110 (72–144)	88	CS	Standard care	—	Stable, worsening, improving	83		CSS	SA, RRB	ASD	U
Macari 2012 [40]	HR	84	12 (NA)	77	CS	Standard care	—	ASD, ATD, TD	12		Total	—	—	P
Messinger 2015 [15]	HR, diag.	1241	24 (24-NA)	58	CS	Standard care	ASD, non-ASD	—	12		—	SA, RRB	—	U
Mosconi 2009 [41]	Diag.	53	31 (18–35)	92	CS	Standard care	—	—	24	26/53	—	—	ASD	P
Ozonoff 2014 [20]	HR	294	7 (NA)	NA	CS	Standard care	—	ASD, ATD, TD	18		—	SA	—	U
Ray-Subramanian 2012 [42]	Diag.	115	31 (24–36)	84	CS	Standard care	—	—	13		—	RRB	ASD	P
Richler 2010 [43]	Diag.	214	24 (24–24)	80	CS	Standard care	—	—	84		—	—	ASD, AD	P
Shumway 2012 [44]	Diag.	89	49 (24–144)	87	CS	Standard care	—	—	17		Total	—	—	U
Solomon 2014 [45]	Diag.	128	50 (32–71)	82	RCT	Carer training, standard care	—	—	12		—	—	ASD	P
Szatmari 2015 [46]	Diag.	406	40 (NA)	84	CS	Standard care	—	—	35		CSS	—	—	P
Thurm 2015 [47]	Diag.	70	41 (12–60)	81	CS	Standard care	—	Minimally verbal, phrase speech	27		CSS	SA, RRB	—	P
Turner 2007 [48]	Diag.	48	29 (24–35)	NA	CS	Standard care	—	—	24		—	—	ASD, AD	P
van Daalen 2009 [16]	HR	131	26 (NA)	85	CS	Standard care	AD, PDD-NOS, non-ASD		19		—	SA, RRB	ASD, AD	P
Venker 2014 [49]	Diag.	127	31 (23–39)	87	CS	Standard care	—	—	36		CSS	—	—	P
Vivanti 2014 [50]	Diag.	57	41 (18–60)	88	CS	Specific, standard care	—	—	12		CSS	SA, RRB	—	P
Zachor 2007 [51]	Diag.	39	28 (22–34)	95	RCT	Specific, standard care	—	—	12		—	SA, RRB	ASD, AD	P

NA–not available.

1 Listing only those relevant to this review.

2 CS–prospective cohort study; RCT–randomised controlled trial.

3 Our categorisation; see main text for descriptions.

4 AD–autistic disorder; ASD–autism spectrum disorder; ATD–atypical development; IQ–intelligence quotient; PDD-NOS–pervasive developmental disorder not otherwise specified; TD–typical development

5 P–published data only; U–unpublished and published data.

6 Diag.–diagnosed; HR–high risk.

7 Subgroups from retrospective classifications were pooled in the meta-analysis to avoid inflated heterogeneity estimates.

8 CSS–calibrated severity score.

9 SA–social affect (incl. subscales communication and reciprocal social interaction in earlier ADOS versions); RRB–repetitive and restricted behaviour.

### Quality of studies

Many studies did not specify attrition rates and were defined as of uncertain quality and risk of bias. Four of the five RCTs and 10 of the 35 prospective cohort studies were rated as good quality (Table 2).

**Table 2:** Quality of included studies.

Study	Quality	Design type	Blinded pre/post	Attrition (%)
Aldred 2004 [25]	Good	RCT	Yes	12%
Dawson 2010 [27]	Good	RCT	Yes	10%
Green 2010 [31]	Good	RCT	Yes	4%
Howlin 2007 [35]	Low	RCT	No	1%
Solomon 2014 [45]	Good	RCT	Yes	12%
Akshoomoff 2004 [23]	Good	CS	Yes	9%
Anderson 2014 [11]	Low	CS	No	47%
Ben Itzchak 2009 [17]	Uncertain	CS	No	Not reported
Brian 2008 [26]	Uncertain	CS	Yes	Not reported
Chawarska 2009 [18]	Uncertain	CS	No	Not reported
Chawarska 2014 [19]	Uncertain	CS	Not reported	Not reported
Dereu 2012 [28]	Good	CS	Not reported	5%
Di Renzo 2015 [21]	Uncertain	CS	Not reported	Not reported
Freitag 2012 [29]	Uncertain	CS	No	Not reported
Gammer 2015 [22]	Good	CS	Not reported	2%
Gotham 2012 [30]	Uncertain	CS	Not reported	Not reported
Guthrie 2013 [32]	Uncertain	CS	No	Not reported
Gutstein 2007 [33]	Low	CS	Not reported	25%
Hobson 2015 [34]	Uncertain	CS	Yes, partly	Not reported
Kleinman 2008 [12]	Low	CS	No	Not reported
Klintwall 2015 [36]	Good	CS	Not reported	12%
Landa 2013 [13]	Good	CS	22	2%
Lerna 2014 [24]	Low	CS	Yes	22%
Lord 2006 [37]	Good	CS	Not reported	10%
Lord 2012 [38]	Uncertain	CS	No	Not reported
Louwerse 2015 [39]	Low	CS	Yes	26%
Macari 2012 [40]	Uncertain	CS	Yes	Not reported
Messinger 2015 [15]	Good	CS	Not reported	14%
Mosconi 2009 [41]	Low	CS	Not reported	49%
Ozonoff 2014 [20]	Uncertain	CS	Not reported	Not reported
Ray-Subramanian 2012 [42]	Uncertain	CS	Not reported	Not reported
Richler 2010 [43]	Uncertain	CS	No info	Not reported
Shumway 2012 [44]	Low	CS	Indep assessments	56%
Szatmari 2015 [46]	Low	CS	Not reported	30%
Thurm 2015 [47]	Uncertain	CS	Not reported	Not reported
Turner 2007 [48]	Good	CS	Not reported	17%
van Daalen 2009 [16]	Uncertain	CS	Not reported	Not reported
Venker 2014 [49]	Good	CS	Not reported	9%
Vivanti 2014 [50]	Good	CS	No	2%
Zachor 2007 [51]	Uncertain	CS	Not reported	Not reported

Studies are listed by design type. CS–prospective cohort study; RCT–randomised controlled trial.

### Stability of autism spectrum disorder over time

#### Overall severity of autism symptoms

**Fig 2:**
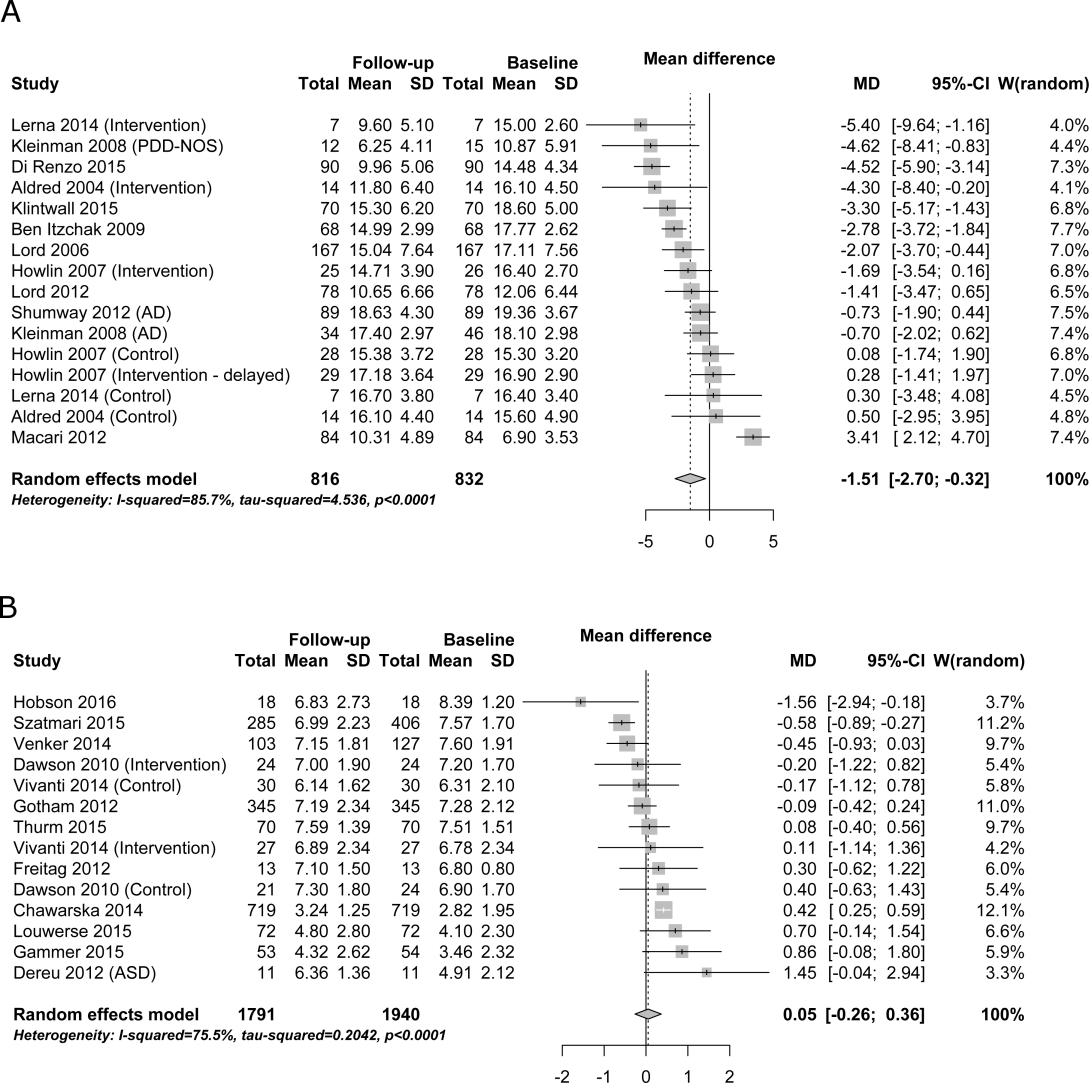
Overall severity of autism symptoms. Panel a–ADOS total scores. Panel b–Calibrated Severity Scores. MD–mean difference (difference in points on the scale from baseline to follow-up).

We performed two separate meta-analyses of overall severity of autism symptoms for ADOS total scores and CSS. There was high heterogeneity between studies for severity of autism symptoms both as expressed in ADOS total scores (I^2^ = 85.6%; Fig 2A) and in CSS (I^2^ = 75.5%; Fig 2B). Significant changes were observed for severity of autism symptoms in ADOS total scores over time (MD = -1.51, 95% CI -2.70 to -0.32). No significant changes were observed for severity of autism symptoms in CSS over time (MD = 0.05, 95% CI -0.26 to 0.36).

#### Severity of autism symptom subdomains

**Fig 3:**
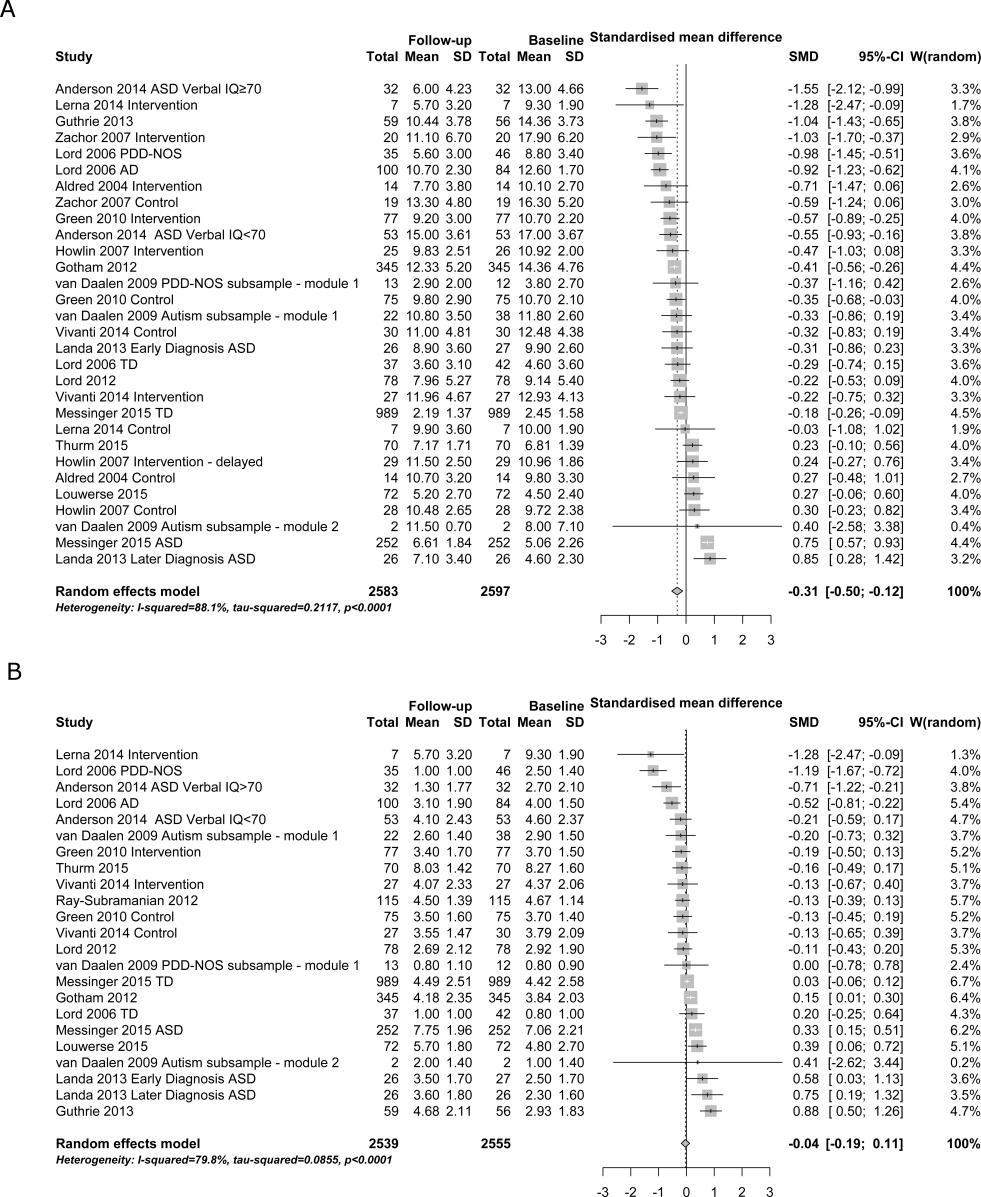
Severity of autism symptom subdomains. Panel a–social affect. Panel b–restricted and repetitive behaviour. MD–mean difference (difference in points on the scale from baseline to follow-up).

The ADOS social affect subdomain showed high heterogeneity between studies (I^2^ = 88.1%) with a significant improvement of medium effect size over time (SMD = -0.31, 95% CI -0.50 to -0.12; Fig 3A), while the ADOS restricted and repetitive behaviour subdomain showed high heterogeneity between studies (I^2^ = 79.8%) with no significant improvement over time (SMD = -0.04, 95% CI -0.19 to 0.11; Fig 3B).

#### Proportion meeting diagnostic criteria

**Fig 4:**
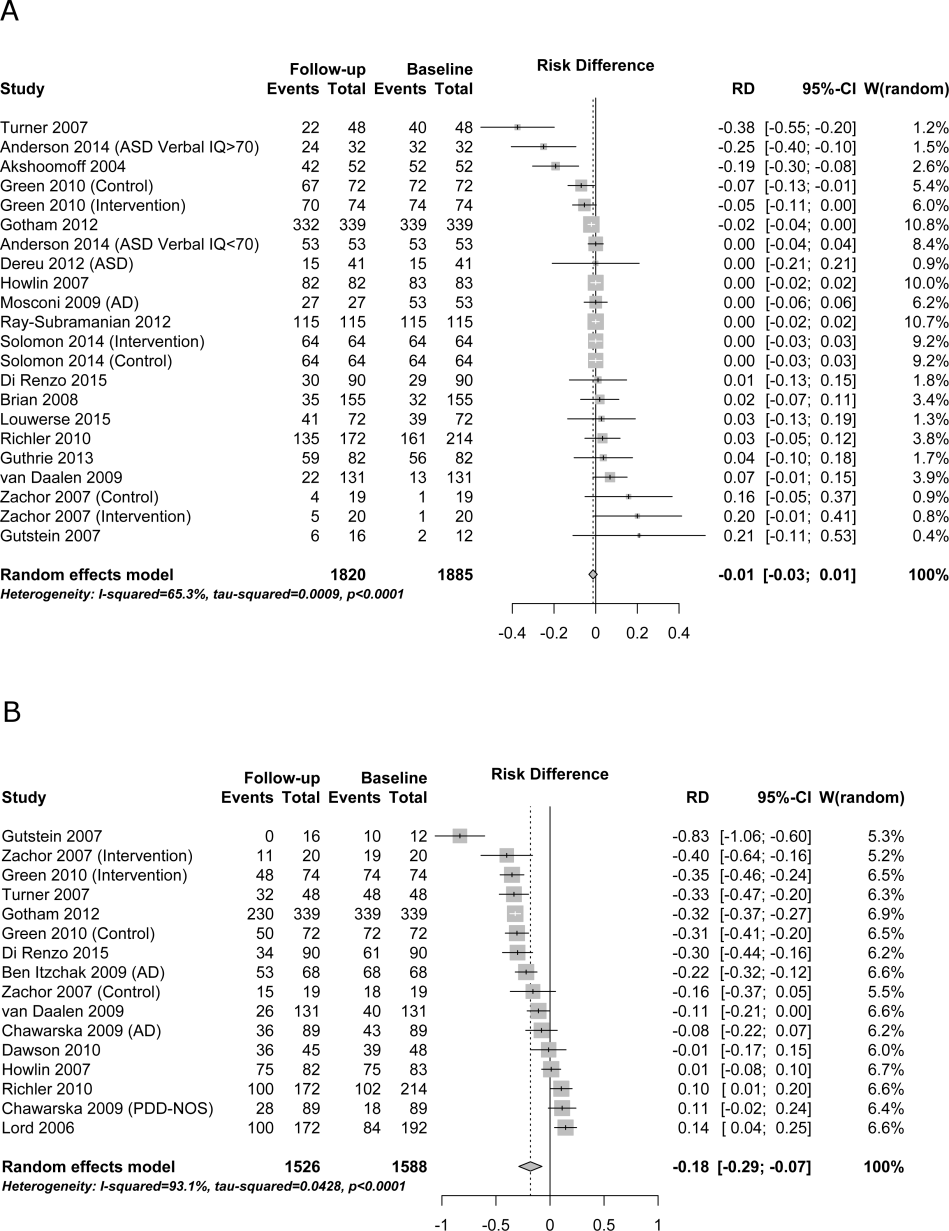
Proportion meeting diagnostic criteria. Panel a–autism spectrum disorder. Panel b–autism. RD–risk difference (difference in percentage of participants meeting the cut-off from baseline to follow-up).

Heterogeneity between studies was high (I^2^ ≥ 50%) for both autism (I^2^ = 93.1%; Fig 4B) and autism spectrum disorder (I^2^ = 65.3%; Fig 4A). Significant changes were observed for meeting autism diagnostic criteria over time (RD = -0.18, 95% CI -0.29 to -0.07; Fig 4B). This risk difference of -0.18 means that 18% of children did not meet the autism criteria at follow-up; numbers meeting autism spectrum disorder criteria at baseline and follow-up, along with the observed totals in each study, are shown in Fig 4B. No changes were observed for meeting autism spectrum disorder criteria over time (RD = -0.01, 95% CI -0.03 to 0.01; Fig 4A).

### Subgroup analyses

Subgroup analyses were conducted to examine differences attributable to type and age of participants, type of intervention, and duration of follow-up. An overview of the results is given in Table 3.

**Table 3:** Random effects meta-analyses and linear mixed-effects meta-regression models.

**Categorical predictor: Type of participants**						
**Outcome (statistic used)**	**P-value**	**Predictor level**	**Number of samples**	**Number of participants**	**Estimate (95% CI)**	**Residual heterogeneity (I**^**2**^**)**
ADOS Total (MD)	0.340	diagnosed	13	503	-1.88 (-2.92 to -0.85)	74%
		high risk	3	329	0.02 (-3.75 to 3.79)	94%
ADOS Calibrated Severity Score (MD)	0.005 **	diagnosed	11	1156	-0.15 (-0.42 to 0.13)	48%
		high risk	3	784	0.56 (0.15 to 0.97)	23%
ADOS Social Affect Total (SMD)	0.136	diagnosed	21	1250	-0.23 (-0.50 to 0.04)	89%
		high risk	9	1347	-0.53 (-0.83 to -0.24)	83%
ADOS Restr. and Rep. Beh. Total (SMD)	0.560	diagnosed	14	1208	0.01 (-0.17 to 0.18)	73%
		high risk	9	1347	-0.10 (-0.43 to 0.22)	86%
Autism spectrum cut-off (RD)	0.040 *	diagnosed	18	1476	-0.02 (-0.04 to 0.00)	74%
		high risk	4	409	0.04 (-0.01 to 0.10)	0%
Autism cut-off (RD)	0.098	diagnosed	14	1265	-0.21 (-0.32 to -0.10)	92%
		high risk	2	323	0.02 (-0.23 to 0.27)	91%
**Continuous predictor: Age at baseline (years)**						
**Outcome (statistic used)**	**P-value**		**Number of samples**	**Number of participants**	**Regression coefficient (95% CI)**	**Residual heterogeneity (I**^**2**^**)**
ADOS Total (MD)	0.774		16	832	-0.08 (-0.64 to 0.48)	86%
ADOS Calibrated Severity Score (MD)	0.922		14	1940	-0.01 (-0.17 to 0.16)	74%
ADOS Social Affect Total (SMD)	0.204		30	2597	0.07 (-0.04 to 0.17)	88%
ADOS Restr. and Rep. Beh. Total (SMD)	0.773		23	2555	-0.01 (-0.11 to 0.08)	81%
Autism spectrum cut-off (RD)	0.925		22	1885	0.00 (-0.01 to 0.01)	67%
Autism cut-off (RD)	0.122		16	1588	-0.05 (-0.12 to 0.01)	93%
**Categorical predictor: Type of intervention**						
	**P-value**	**Predictor level**	**Number of samples**	**Number of participants**	**Estimate (95% CI)**	**Residual heterogeneity (I**^**2**^**)**
ADOS Total (MD)	0.001 **	standard care	8	500	-0.52 (-2.16 to 1.13)	84%
		carer training	4	97	-0.83 (-2.33 to 0.66)	50%
		specific	4	235	-3.57 (-4.63 to -2.52)	41%
ADOS Calibrated Severity Score (MD)	0.067	standard care	10	1858	0.12 (-0.23 to 0.47)	81%
		carer training	1	18	-1.56 (-2.94 to -0.18)	—
		specific	3	64	0.08 (-0.52 to 0.68)	0%
ADOS Social Affect Total (SMD)	0.416	standard care	22	2369	-0.28 (-0.51 to -0.06)	90%
		carer training	5	174	-0.23 (-0.65 to 0.19)	70%
		specific	3	54	-0.74 (-1.41 to -0.07)	59%
ADOS Restr. and Rep. Beh. Total (SMD)	0.383	standard care	20	2444	0.00 (-0.16 to 0.15)	81%
		carer training	1	77	-0.19 (-0.50 to 0.13)	—
		specific	2	34	-0.58 (-1.67 to 0.52)	66%
Autism spectrum cut-off (RD)	0.873	standard care	17	1562	-0.02 (-0.04 to 0.01)	71%
		carer training	4	233	-0.01 (-0.03 to 0.02)	39%
		specific	1	90	0.01 (-0.13 to 0.15)	—
Autism cut-off (RD)	0.510	standard care	9	1083	-0.15 (-0.30 to 0.00)	94%
		carer training	3	169	-0.38 (-0.77 to 0.02)	97%
		specific	4	336	-0.12 (-0.30 to 0.05)	87%
**Continuous predictor: Duration of follow-up (years)**						
**Outcome (statistic used)**	**P-value**		**Number of samples**	**Number of participants**	**Regression coefficient (95% CI)**	**Residual heterogeneity (I**^**2**^**)**
ADOS Total (MD)	0.341		16	832	-0.36 (-1.09 to 0.38)	85%
ADOS Calibrated Severity Score (MD)	0.879		14	1940	-0.02 (-0.22 to 0.19)	73%
ADOS Social Affect Total (SMD)	0.025 *		30	2597	-0.05 (-0.09 to -0.01)	86%
ADOS Restr. and Rep. Beh. Total (SMD)	0.042 *		23	2555	-0.03 (-0.07 to 0.00)	79%
Autism spectrum cut-off (RD)	0.449		22	1885	0.00 (-0.01 to 0.00)	67%
Autism cut-off (RD)	0.164		16	1588	0.04 (-0.02 to 0.09)	93%

MD–mean difference; RD–risk difference; SMD–standardised mean difference.

* p < .05

** p < .01.

#### Type of participants

Participant type was a significant predictor of ADOS CSS (p = 0.005) and autism spectrum disorder (ADOS instrument classification) cut-off (p = 0.04; first part of Table 3). Significant deterioration by about half a point on the ADOS CSS was observed for the subgroup at high risk (MD 0.56, 95% CI 0.15 to 0.97, residual heterogeneity I^2^ = 23%), whereas those with a diagnosis at baseline did not change (MD -0.15, 95% CI -0.42 to 0.13; Table 3). For the proportion meeting the autism spectrum disorder (ADOS instrument classification) cut-off, marginal improvement was observed for those with a diagnosis at baseline (RD -0.02, 95% CI -0.04 to 0.00; i.e. a reduction by 2 percentage points; but with high residual heterogeneity of I^2^ = 74%), compared to no significant change in the high risk subgroup (RD 0.04, 95% CI -0.01 to 0.10; i.e. a non-significant increase by 4 percentage points; Table 3). Forest plots of the meta-analyses where this predictor was significant are available in the supplemental material (S1 and S2 Figs).

#### Participants’ age

There were no statistically significant effects of participants’ age on ADOS total score, ADOS CSS, ADOS SA, ADOS RRB, ASD cut-offs, and autism cut-offs (second part of Table 3).

#### Type of intervention

The type of intervention predicted changes on ADOS total scores (p = 0.001), but not on any other measure (third part of Table 3). Significant improvements were observed in those who received a specific intervention (MD -3.57, 95% CI -4.63 to -2.52, I^2^ = 41%; i.e. an improvement of about four points on the ADOS total scale) compared to no change in children who received standard care (MD -0.52, 95% CI -2.16 to 1.13) or carer training (MD -0.83, 95% CI -2.33 to 0.66; Table 3) on this scale.

#### Duration of follow up

**Fig 5:**
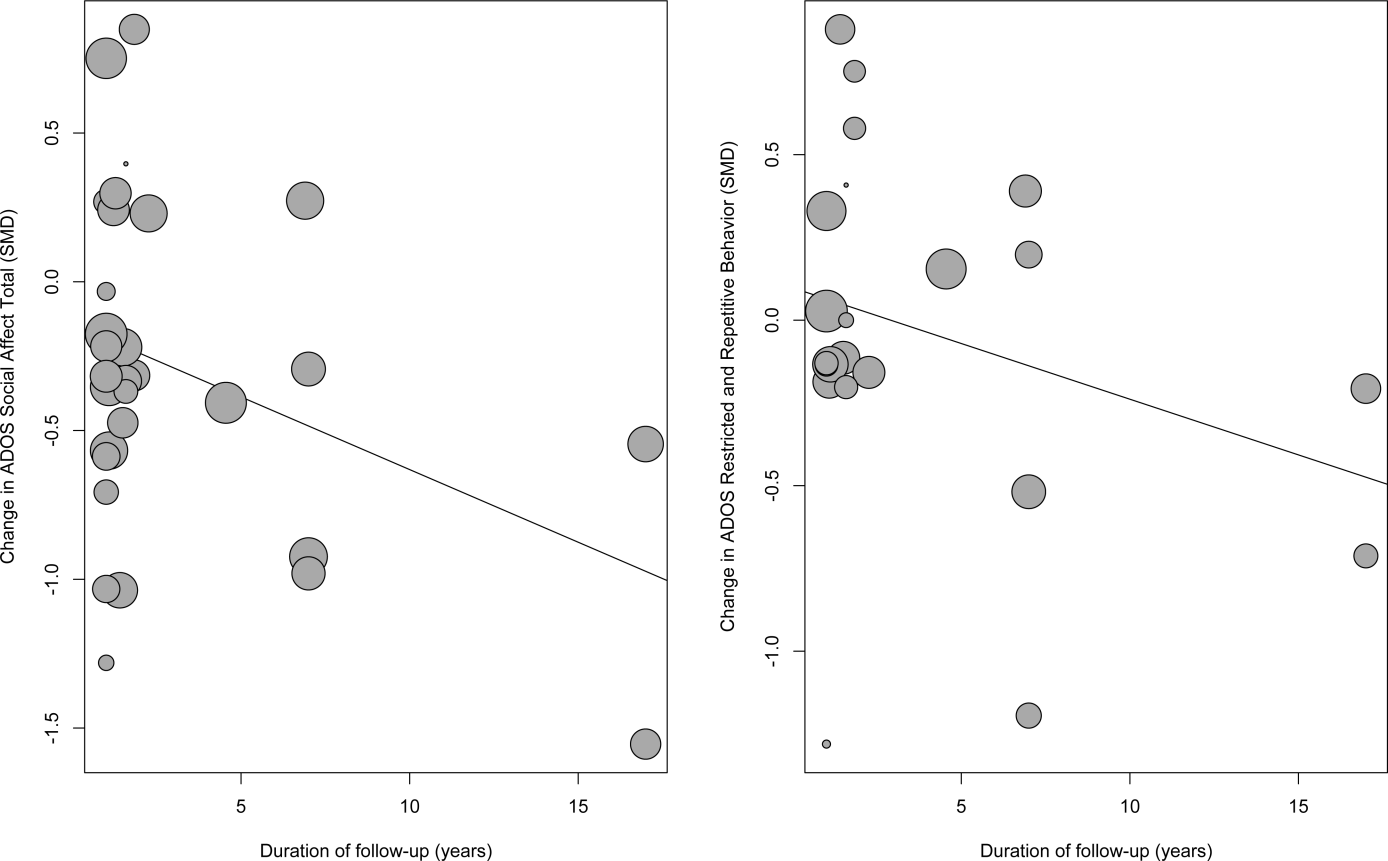
Results of meta-regression analyses. Shows magnitude of changes as a function of follow-up duration. Circles represent individual studies, with circle sizes representing sample sizes.

Longer duration of follow-up was associated with greater improvements in the two ADOS subscales, but not on the other measures (last part of Table 3). For the ADOS social affect, improvement increased by 0.05 points per year (p = 0.025; regression coefficient -0.05, 95% CI -0.09 to -0.01), corresponding to half a point over ten years. ADOS restricted and repetitive behaviour scores improved by 0.03 points per year (p = 0.042; regression coefficient -0.03 per year, 95% CI -0.07 to 0.00; Table 3), or one-third of a point over ten years. The magnitude of change was small even after ten years or more, and there were only few samples with long follow-up duration (Fig 5).

## Discussion

Although autism spectrum disorder/autism diagnosis and tracking autism symptoms over time has long been of interest [5,52,53], to our knowledge this is the first comprehensive meta-analysis examining the temporal stability of autism spectrum disorder (as defined by ADOS instrument classification) and severity of autism symptoms using the ADOS. We found no change in ADOS scores across time as measured by the most phenotypically stable autism core symptom measure; the CSS. There was a minor but statistically significant change in ADOS total scores (1.51 point reduction across up to 15 years) and a minor reduction in the subdomain of social affect symptoms (-0.31 points). There was an 18% reduction (risk difference -0.18, 95% CI -0.29 to -0.07; Fig 4B) of children meeting the autism criteria according to ADOS total scores, but no change in overall autism spectrum disorder prevalence, suggesting that some children move from autism to other autism spectrum disorder diagnoses. Although not salient in the overall analyses, sub-group analyses on ADOS total scores also showed that a net 2% fulfilling autism spectrum disorder cut-off diagnosis (ADOS instrument classification) at baseline lost their diagnosis (risk difference -0.02, 95% CI -0.04 to 0.00; Table 3, autism spectrum cut-off/participant type diagnosed). The only significant change of sub-group analyses for CSS was a deterioration of individuals at risk compared to already diagnosed children. This is also the most robust finding with low heterogeneity (23%).

The scientific community view ASD as a neurodevelopmental disorder, similar to other neurodevelopmental disorders such as intellectual disability and language disorders. The very name—pervasive developmental disorder—further highlights this view. When it comes to treatment research however, ASD is approached quite differently from intellectual disability; interventions are often evaluated using measures of core autism symptoms such as the ADOS. Intervention programs for intellectual disability on the other hand rarely aim for improvement of the intellectual disability but rather at improved outcome, functioning and well-being. Although the ADOS is probably not a sensitive measure of change and thus may underestimate possible change, our findings do support the view of ASD as a stable neurodevelopmental disorder at group level. Consequently, intervention studies should focus less on core autism symptoms in favour of areas more relevant to the affected individuals such as quality of life, general functioning and outcome.

The relative improvement of ADOS total scores versus no improvement in CSS may indicate that studies using ADOS total scores as an outcome measure may achieve artificial improvements due to changes between ADOS modules and general development rather than a true change of core autism symptoms [6]. The rationale for creating the CSS was to provide a more stable measure for longitudinal studies. This developmental bias will have a particular impact in studies of young children, where there are more frequent shifts in ADOS modules. Most included studies only reported either CSS or ADOS total scores, making direct comparisons across all studies impossible. Findings highlight a crucial role for ADOS CSS as a measure of severity of autism symptoms in being less sensitive to phenotypic and environmental changes than ADOS total scores. The high heterogeneity for ADOS total scores analyses (85.7%; Fig 2A) may indicate that the raw total scores are significantly influenced by individual phenotypical characteristics and demographics and may therefore be a problematic measure of autism symptom severity [6,54]. As intended, CSSs are more uniformly distributed within diagnostic categories (autism, PDD-NOS, non-autism spectrum, or typical development) and across assessment modules than are total scores [55].

Research on change in autism symptoms subdomains over time is still emerging. Our results indicate that individuals with autism symptoms may show some change in social affect (such as pointing/showing, gestures, eye contact, joint attention, social overtures, and others), but not in restricted and repetitive behaviours (such as unusual sensory interest in play, self-injurious behaviours, stereotyped behaviours). However, the change in social affect was small (-0.31 points) and may be an artefact of applying different modules across development, as CSSs were stable. The lack of change in both raw scores and CSS scores in the RRBI subdomain is likely due to the lesser impact of module change noted for the RRBI domain as already shown by Hus et al. [56]. Our results are in line with previous research by Hus et al. showing that restricted and repetitive behaviours are more persistent over time and less sensitive to children’s phenotypic characteristics compared to social affect [56]. Furthermore, most therapeutic interventions target primarily communication and social skills development while addressing restricted and repetitive behaviours to a lesser degree [57].

Children receiving specific intervention had an improvement in their ADOS total scores over time, consistent with previous meta-analyses of the positive effects of early specific intervention for children with autism spectrum disorder [58]. However, the studies investigating specific interventions using CSS as outcome measure did not show such improvement, which again could indicate an artificial effect due to module changes. The sub-analysis of specific interventions with ADOS total scores as the outcome measure included only four studies (234 participants) of which none were RCTs [17,21,24,36] and only one was of good quality [36]. Furthermore, we could not confirm a significant impact of carer training on reducing autism severity, contrary to the results in a previous systematic review on parent-mediated early intervention for young children [59], possibly due to the limited number of studies targeting parent/carer in our meta-analysis (4 RCTs, 97 participants). These results therefore need to be interpreted with caution. The sub-analysis of type of participants revealed that participants at risk of autism spectrum disorder (mostly sibling studies) showed deterioration in overall severity of autism symptoms as expressed in CSS compared to individuals already diagnosed with autism spectrum disorder. This might be due to the significant number of autism spectrum disorder siblings that progress to full-blown autism spectrum disorder in the course of the first three years of life [60]. The final set of sub-analysis showed that duration of follow-up plays a significant role in predictors of severity of social affect and restricted and repetitive behaviour. Symptoms tended to improve more with increasing follow-up duration, however the findings are limited by the fact that only one study (with two samples) had a follow-up of more than seven years [11]. The improvement was small, with predicted ten-year change in social affect and restricted and repetitive behaviour being less than one point. The sub-analysis of participants’ age indicated that participant age at baseline did not come out as a moderating factor of outcome at follow-up, consistent with autism being a neurodevelopmental disorder and fairly stable in its expression.

### Limitations

This review was limited by the quality of reporting in the studies included. Results reported in different formats (raw scores, CSS, subscales, total scores) and without indication of ADOS version posed a major challenge in extracting data and impacted the meta-analyses. We contacted authors to request missing data or clarification, and successfully obtained requested data in some cases.

The limited number of high quality studies both for intervention and follow-up further limit the strength of the evidence. Most commonly, studies only reported on children that were included at both baseline and follow-up, while failing to detail the total number of children available at baseline. Attrition may introduce bias of uncertain direction as parents of children with either a very good outcome or very poor outcome may be less likely to engage in a follow-up study. Moreover, blinding is difficult to achieve and remains a factor in determining the accuracy of results for intervention studies.

It may also be that some studies reported on overlapping samples. Unfortunately, it was not possible from the papers to identify such overlap unambiguously. However, even if samples actually overlapped, they were not included in the same meta-analyses as long as the studies reported different ADOS measures. Therefore, while overlapping samples may have distorted descriptive statistics such as the total number of participants included in the review, they seem unlikely to have led to incorrect estimates in meta-analyses.

The included studies had high levels of clinical heterogeneity precluding firm conclusions, with variations in the type of the ADOS applied (ADOS/ADOS-G/ADOS-2/ADOS toddler version) and different versions available in different countries. More research is needed to systematically explore such variations and learn from such comparisons. Further analysing the various clinical subdivisions made in several studies (Table 1) would be relevant, but was out of scope of this review and would require individual participant data, which from most studies were not available.

While some studies included information about the participants’ cognitive skills, many did not. Therefore, it was not possible to explore cognitive development as a predictor of outcome.

The evidence is limited to the childhood period as there were no studies of adults or following children into adulthood in our review. One study [60] that followed the diagnostic stability of Asperger Syndrome did find that a minority (22%) of children did not fulfil the criteria in adulthood. However, that study did not use the ADOS to evaluate changes and therefore did not meet our stringent criteria.

### Implications for research and practice

The temporal stability of autism spectrum disorder/autism diagnosis and core symptom severity as measured by the ADOS puts solid evidence behind the definition of autism spectrum disorder as a neurodevelopmental condition/trait similar to intellectual disability, learning disability or social learning disability. While only five studies were RCTs investigating intervention outcomes, all studies included children receiving some form of intervention. Despite this fact, overall outcomes at follow-up did not show any significant change in core autism symptoms. These results indicate a need to redefine the focus of autism spectrum disorder intervention and support. Rather than targeting core autism symptoms, our results suggest that intervention studies should focus on other measures of outcome such as quality of life and adaptive functioning.

There is a great need for rigorously designed studies including larger sample sizes with transparent and complete reporting of study results. Very few intervention studies were randomized and applied blinding of assessment at outcome, creating a high risk of bias. Future studies should aim to publish or make available results both as expressed in ADOS total scores, raw scores and CSS, and they should also specify which ADOS version is being used. Studies wanting to claim treatment gains from intervention should be requested to report CSS to avoid artificial effects of module changes.

The great stability of the ADOS scores and the limited range suggests, as has been indicated previously [56], that the ADOS is not a good measure of change. ASD research is in great need of better autism measures, including also biomarkers and such work is ongoing [61]. In the meantime, intervention studies should strive to examine alternative measures such as quality of life and daily function. Finally, greater efforts are needed to ensure longer follow-up, and studies into adulthood to assess the long-term outcome of an autism spectrum disorder/autism diagnosis.

## Conclusions

Our findings indicate a remarkable stability of overall autism severity and autism symptoms over time across childhood on a group level. On a sub-group level, the only robust finding was that children at high risk deteriorated over time (observed in CSS, I^2^ = 23%). Using ADOS total scores, 18% of participants shifted from autism to autism spectrum disorder diagnosis, however the overall autism spectrum disorder prevalence was unchanged. The results confirmed that ADOS CSS is one of the most robust (regardless of age, cognitive ability or language) measures of autism severity available, and seems to measure autism symptoms in a similar way as intellectual quotient measure cognitive ability. As evidenced by other studies, individual trajectories do change over time, but at the group level the ADOS CSS are stable across childhood. In addition to confirming the stability of autism spectrum disorder over time, this review highlights a need for improved transparency in research reporting and for rigorously designed studies including larger sample sizes with complete reporting of study results to enable comparisons across studies.

## Supporting information

S1 Checklist PRISMA-P checklist. Lists the reporting of important methodological details in the review protocol.
(DOCX)

S2 Checklist PRISMA checklist. Lists the reporting of important methodological details in the final review.
(DOC)

S1 Protocol Protocol for systematic review. Prespecified protocol as used in the review process.
(DOCX)

S1 Fig Overall severity of autism symptoms (Calibrated Severity Scores), split by participant type. (TIF)

S2 Fig Proportion meeting autism spectrum disorder criteria (ADOS instrument classification), split by participant type. (TIF)

S1 Dataset Data and syntax. Contains data used for meta-analysis and R syntax to reproduce the analyses.
(ZIP)
