# Supplementary material for: Tracing the temporal stability of autism spectrum diagnosis and severity as measured by the Autism Diagnostic Observation Schedule: A systematic review and meta-analysis
Source: PLoS One. 2017 Sep 21;12(9):e0183160. doi: 10.1371/journal.pone.0183160 (PMC5608197; doi:10.1371/journal.pone.0183160)
Supplement: S1 Protocol — Prespecified protocol as used in the review process. (DOCX) [file pone.0183160.s003.docx]

**Section 1: Administrative information**

***Title***

Tracing the temporal stability of autism spectrum diagnosis and severity using ADOS: protocol for a systematic review

***Authors***

Łucja Bieleninik¹, Maj-Britt Posserud², Monika Geretsegger¹, Grace Thompson³, Cochavit Elefant^4^, Christian Gold¹*****

¹GAMUT – The Grieg Academy Music Therapy Research Centre, Uni Research Health, Bergen, Norway; ²Department of Child and Adolescent Psychiatry, Haukeland University Hospital; Regional Centre for Child and Youth Mental Health and Child Welfare, Uni Research Health, Bergen, Norway; ³Melbourne Conservatorium of Music, The University of Melbourne, Melbourne, Australia; ^4^School for Creative Arts Therapies, University of Haifa, Israel

****Corresponding author***

Christian Gold, GAMUT – The Grieg Academy Music Therapy Research Centre, Uni Research Health, Bergen, Norway, Lars Hilles gt. 19, 5015 Bergen, Norway, [christian.gold@uni.no], +47-97501757.

***Contributions***

ŁB and CG are the guarantors. ŁB, MBP, MG and CG drafted the manuscript. All authors contributed to the development of the selection criteria the risk of bias assessment strategy and data extraction criteria. ŁB developed the search strategy. CG provided statistical expertise. MBP provided expertise on the ADOS instrument. All authors read, provided feedback and approved the final version of protocol.

***Amendments***

The search will be updated toward the end of the review in order to increase a high proportion of eligible studies.

***Sources***

This systematic review received no specific grant from any funding agency in the public, commercial, or not-for-profit sectors. There was no role of sponsor and funder for the review. ŁB, MG and CG were supported by the Research Council of Norway (grant no. 213844).

**Section 2: Introduction**

***Rationale***

The lack of knowledge on the natural progression of autism spectrum disorder (ASD) is one of the many challenges in research. Tracking ASD is hampered by the lack of valid and reliable measures of symptoms across the life-span and developmental level. Few measures currently exist to track the temporal stability of ASD/autism and severity of the core symptoms over time, as several commonly used instruments (e.g. the Autism Behaviour Checklist, the Childhood Autism Rating Scale, the Gilliam Autism Rating Scale) are not independent of phenotypic characteristics such as age, IQ, and language level (Gotham, Pickles, and Lord, 2009). One exception is the Autism Diagnostic Observation Schedule (ADOS) (Lord, Rutter, DiLavore & Risi, 2006) which has different modules tailored to the language level and age of the individual to ensure consistency of autism severity scores across cognitive levels and different age groups from infants to adults. The ADOS-based Calibrated Severity Scores (CSS) has been suggested as the most appropriate measure of outcome for treatment and follow-up studies looking to capture change in symptom severity independent of developmental factors (Gotham, Pickles, and Lord, 2009); however ADOS raw totals have been used for many years and are still commonly used. Little is known about the overall “natural” development of ADOS scores in individuals with autism spectrum disorder, and the magnitude of change that could be expected.

During the protocol stage of our project (July 1 to October 30, 2014), we did a scoping search of the literature. We searched PubMed, PsycInfo, EMBASE, Web of Science, Cochrane Library for a term “Autism Diagnostic Observation Schedule”, with no language, reference type or date restrictions. We included people of all ages with ASD/autism or at risk of having ASD, who were diagnosed and followed up for at least 12 months using the gold-standard ADOS tool. We found just one comprehensive review article (Woolfenden, Sarkozy, Ridley & Williams, 2012) and no meta-analysis that had been published at that time. Woolfenden et al.’s narrative summary based on 23 studies with a total of 1466 participants has demonstrated that autism is a reasonable stable diagnosis before three years of age, however the authors indicated that a significant minority of children will no longer meet diagnostic criteria after a period of follow up, particularly those who were diagnosed with cognitive impairments in their preschool years (Woolfenden et al., 2012).

To date, there has been no meta-analysis focusing on prospective cohort studies addressing the diagnostic stability over time of autism spectrum disorder/autism as measured by the ADOS. The motivation to undertake this study was thus to examine the temporal stability of autism spectrum disorder/autism diagnoses over time as measured by the ADOS and plot longitudinal trajectories of core autism symptom severity using the ADOS. The third aim was to identify possible predictors of change in autism spectrum disorder/autism.

***Objectives***

The objectives of our study are to systematically review the literature for prospective cohort studies addressing the diagnostic stability over time of autism spectrum disorder/autism and prospective studies of intervention effects in order to:

1. evaluate the temporal stability of autism spectrum disorder over time as measured by the ADOS;
2. evaluate the temporal stability of core autism symptom severity: social affect and restricted and repetitive behaviours as measured by the ADOS;
3. track the proportion meeting autism spectrum disorder and autism criteria;
4. identify possible predictors of change in autism spectrum disorder and autism.

**Section 3: Methods**

We undertook a comprehensive search following guidelines outlined in the Preferred reporting items for systematic review and meta-analysis protocols (PRISMA-P) (Shamseer et al., 2015).

***Eligibility criteria***

Studies will be selected according to the criteria outlined below.

1. Participants

We will include individuals of any age (including adults) diagnosed with any autism spectrum disorder as defined in DSM-5 (APA, 2013), or similarly as based on earlier versions of the DSM or the International Classification of Diseases (ICD). This will include childhood autism, atypical autism, Asperger syndrome, and pervasive developmental disorder not otherwise specified (PDD-NOS) and autism spectrum disorder, excluding Rett syndrome. We also aim to include individuals at risk of having autism spectrum disorder (e.g. siblings with autism spectrum disorder or through screening instruments).

1. Baseline assessment

ASD/autism diagnosis at baseline must be measured with a version of the ADOS (ADOS/ADOS-G/ADOS-2/ADOS toddler version) as diagnostic measure. Other standardized tools such as: the Autism Diagnosis Interview Revised (ADI-R), the Childhood Autism Rating Scale (CARS), the Diagnostic Interview for Social and Communication Disorders (DISCO), the Gilliam Autism Rating Scale (GARS), and the Developmental, Dimensional and Diagnostic Interview (3di), can be used in addition.

1. Follow-up assessment

ASD/autism diagnosis at least 12 months later must be measured with a version of the ADOS (ADOS/ADOS-G/ADOS-2/ADOS toddler version) as diagnostic measure. If participants will be evaluated using ADOS more than twice, we will analyse the longest available time span.

1. Type of study

We will include prospective cohort studies addressing the diagnostic stability over time of ASD/autism; or prospective studies of intervention effects (i.e., randomised, non-randomised controlled, or without a control group).

***Information sources***

Review will be done by search eligible studies of electronic databases searching. In order to provide a reasonable breadth and depth on topic relevant trials were identified more than one database. We will search PubMed, PsycInfo , Web of Science, EMBASE, DARE, and the Cochrane Central Register of Controlled Trials (Wiley interface, current issue). To ensure literature saturation, we will scan the reference lists of relevant reviews identified through the search. The literature search will be not restricted to any language, reference type, or year of publication. Unpublished studies such as conference abstracts and dissertation abstracts will be also included.

***Search strategy***

No language, reference type, or year of publication limits will be imposed on the search. The databases (PubMed, PsycInfo, Web of Science, EMBASE, DARE, and the Cochrane Central Register of Controlled Trials) were systematic search using term “Autism Diagnostic Observation Schedule” by one reviewer. As relevant studies are identified, reviewer will check for additional relevant cited and citing articles.

***Study records***

All potentially relevant records will be extracted to EndNote reference management software. At this stage duplicates will be detected and deleted. Duplicate publication detection will be based on author names, location and setting, specific details of the interventions, numbers of participants and baseline data; and date and duration of the study. When uncertainties remained, we contacted authors.

Titles and abstracts of all references identified will be inspected independently by at least two reviewers to exclude obviously irrelevant reports. Anny disagreement will be solved through dissuasion, and consultation with a third reviewer. We will obtain full reports for all titles that appear to meet the inclusion criteria or where there is any uncertainty. Two review authors independently will then screen the full text reports and decide whether these meet the inclusion criteria. We will seek additional information from study authors where necessary to resolve questions about eligibility. We will resolve disagreement through discussion with a third reviewer. We will record the reasons for excluding trials. Neither of the review authors will be blind to the journal titles or to the study authors or institutions.

Data will be independently extracted by two authors. They will confirm accuracy using a shared, piloted data extraction sheet on participant characteristics at baseline (diagnosed vs. high risk, number, age at initial diagnosis using ADOS, gender), clinical subgroups, study design, interventions, age at follow up, attrition, and outcome category (ADOS total vs. CSS, ADOS subscales, autism spectrum disorder/autism cut-offs). We will resolve disagreement through discussion with other reviewers. We will seek additional information from study authors when required.

***Data items***

We will extract information the following information:

- Participant characteristics at baseline as: type of participants (diagnosed vs. high risk), clinical subgroups, age in months (mean and range), gender.
- Study design type: prospective cohort study vs. randomised controlled trial.
- Intervention type: specific vs. carer training vs. standard care
- Follow up: duration in months and attrition rate.
- Outcomes: Total autism severity, autism severity (subdomain social affect and restricted and repetitive behaviour), meeting autism spectrum disorder criteria and meeting autism criteria.

When study results will be split according to clinical characteristics, we will prepare them as follows: we retained subgroups assigned prospectively (e.g. to intervention vs. control, or divisions between diagnostic groups) because they might contain important information on heterogeneity. For studies that separated participants retrospectively (e.g., into improved or not improved or retrospective diagnostic groups), without pooled data available from the paper or the authors, we will pool these subsamples using means and pooled SDs.

***Outcomes and prioritisation***

Meta-analysis will be performed for the following outcomes:

- Total autism severity: either raw scores using any of the published algorithms, or CSS. We will prioritize CSS over raw total scores if both were available for the complete sample.
- Autism severity, subdomain social affect: social affect subtotal, or social+communication total, or (language and) communication domain & social (interaction) domain, or modified scores thereof.
- Autism severity, subdomain restricted and repetitive behaviour: restricted and repetitive behaviour subtotal, or modified score thereof.
- Meeting autism spectrum disorder criteria, based on ADOS cut-off value (i.e. ≥ 4 on the CSS, or ≥ 7 to ≥ 11 in the different modules of ADOS raw totals [6]; differing between earlier and later ADOS versions).
- Meeting autism criteria, based on ADOS cut-off value (i.e. ≥ 6 on the CSS, or ≥ 9 to ≥ 16 in the different modules of ADOS raw totals [6]; differing between earlier and later ADOS versions).

***Risk of bias individual studies***

In order to evaluate study quality we will collect information on design type (RCTs versus prospective cohort studies), assessor blinding to pre/post ADOS evaluation and attrition rate. Attrition will be rated as low when overall attrition is more than 20%. RCTs will be rated as “low quality” if there was no blinding. Studies failing to report the total number assessed at baseline and RCT studies failing to report blinding status will be labelled as of “uncertain quality”.

***Data synthesis***

For continuous variables, we will use primarily weighted mean differences (MDs). For ADOS raw scores (which have similar but not identical meaning due to various adaptations, versions, and modules used), we also will examine whether using standardized mean differences (SMDs) affected results. This will be not necessary for ADOS CSS, where the scores have the same meaning across ADOS modules. The ADOS subscales social affect and restricted and repetitive behaviour will be analysed using SMD as they were reported in various forms.

Dichotomous variables will be evaluated using risk differences (RDs) because they are straightforward to interpret as percentage point change.

Statistical heterogeneity will be tested using the statistic I^2^ (0% to 40%: might not be important; 30% to 60%: may represent moderate heterogeneity; 50% to 90%: may represent substantial heterogeneity; 75% to 100%: considerable heterogeneity). We will calculate both fixed-effects and random-effects meta-analyses, but if statistical heterogeneity for all main analyses is observed (I^2^ >=50%), the random effects model will be chosen. As potential predictor variables, we will analyse initial age, initial diagnosis (diagnosed vs. high-risk), duration of follow-up, and type of intervention (specific vs. carer training vs. standard care), using random-effects meta-analysis and meta-regression. Each outcome will be combined and calculated using the statistical software R version 3.3.1 (www.r-project-org) and R package meta.

Subgroup analyses will be used to explore possible sources of heterogeneity including the following categorical predictors/predictor levels

(a) intervention; specific vs. carer training vs. standard care,

(b) divisions between diagnostic groups participant; having a diagnosis vs. high risk.

Subgroup analyses were performed for the following continuous predictors:

(c) duration of follow up,

(d) participants’ age at baseline.

Studies that will separate participants retrospectively (e.g., into improved or not improved; or retrospective diagnostic groups), without pooled data on these subsamples will be pooled using means and pooled SDs.

***References***

1. American Psychiatric Association. Diagnostic and Statistical Manual of Mental Disorders (DSM-5). 5th Edition. Arlington, VA: American Psychiatric Publishing, 2013.
2. Gotham K, Pickles A, Lord C. Standardizing ADOS scores for a measure of severity in autism spectrum disorders. J Autism Dev Disord. 2009 May;39(5):693-705. doi: 10.1007/s10803-008-0674-3. Epub 2008 Dec 12.
3. Lord C, Rutter M, DiLavore PS, Risi, S. Autism Diagnostic Observation Schedule (ADOS). Los Angeles: Western Psychological Services; 2006.
4. Shamseer L, Moher M, Clarke M, Ghersi D, Liberati A, Petticrew M, Shekelle P, Stewart LA, the PRISMA-P Group. Preferred reporting items for systematic review and meta-analysis protocols (PRISMA-P) 2015: elaboration and explanation. BMJ. 2014;349:g7647 doi: 10.1136/bmj.g7647
5. Woolfenden S, Sarkozy V, Ridley G, Williams K. A systematic review of the diagnostic stability of autism spectrum disorder. Res Autism Spectr Disord. 2012 Jan-Mar;6(1):345-354.
