# Supplementary figures and images for: Tracing the temporal stability of autism spectrum diagnosis and severity as measured by the Autism Diagnostic Observation Schedule: A systematic review and meta-analysis
Source: PLoS One. 2017 Sep 21;12(9):e0183160. doi: 10.1371/journal.pone.0183160 (PMC5608197; doi:10.1371/journal.pone.0183160)

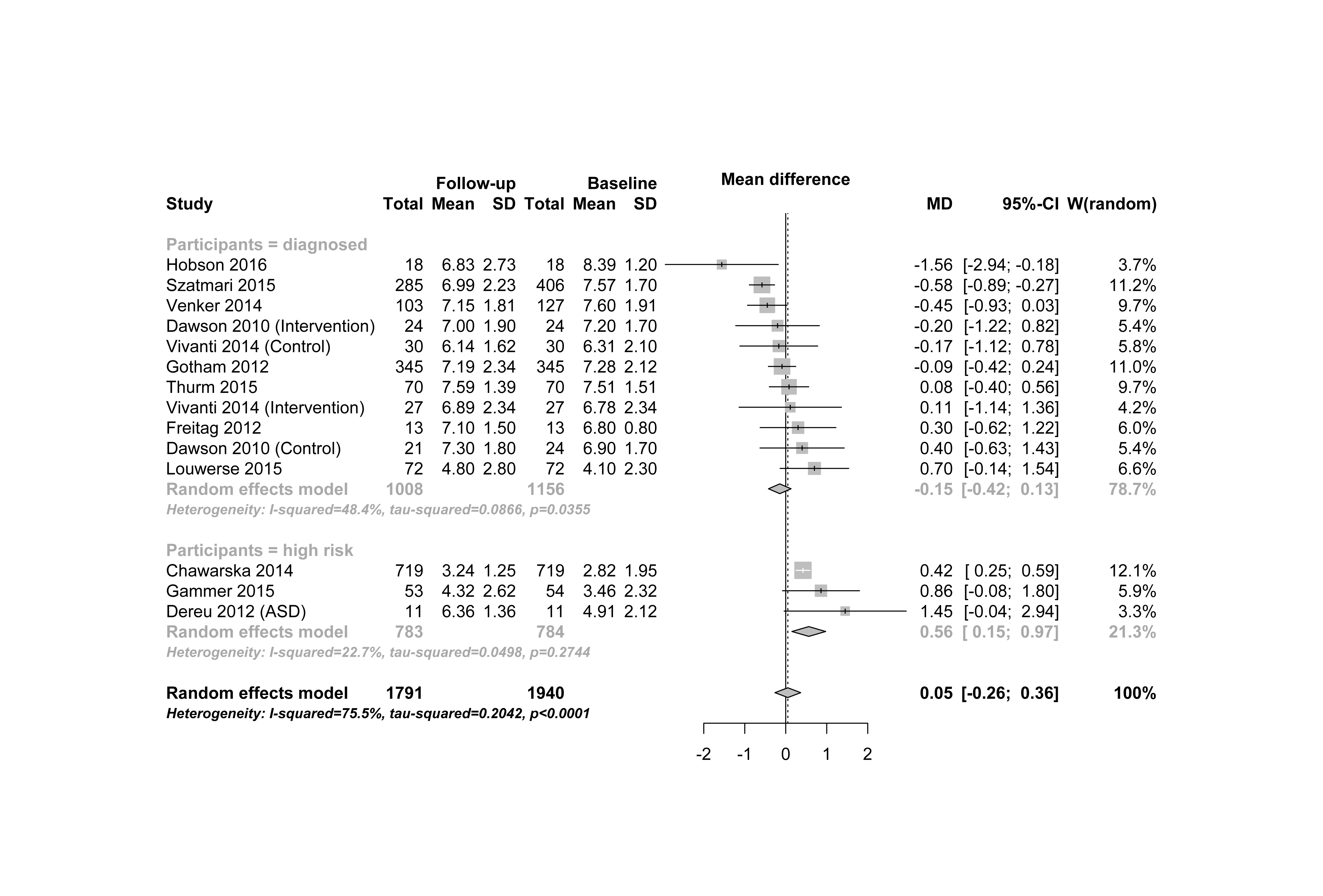

Supplement: S1 Fig — (TIF) [file pone.0183160.s004.tif]

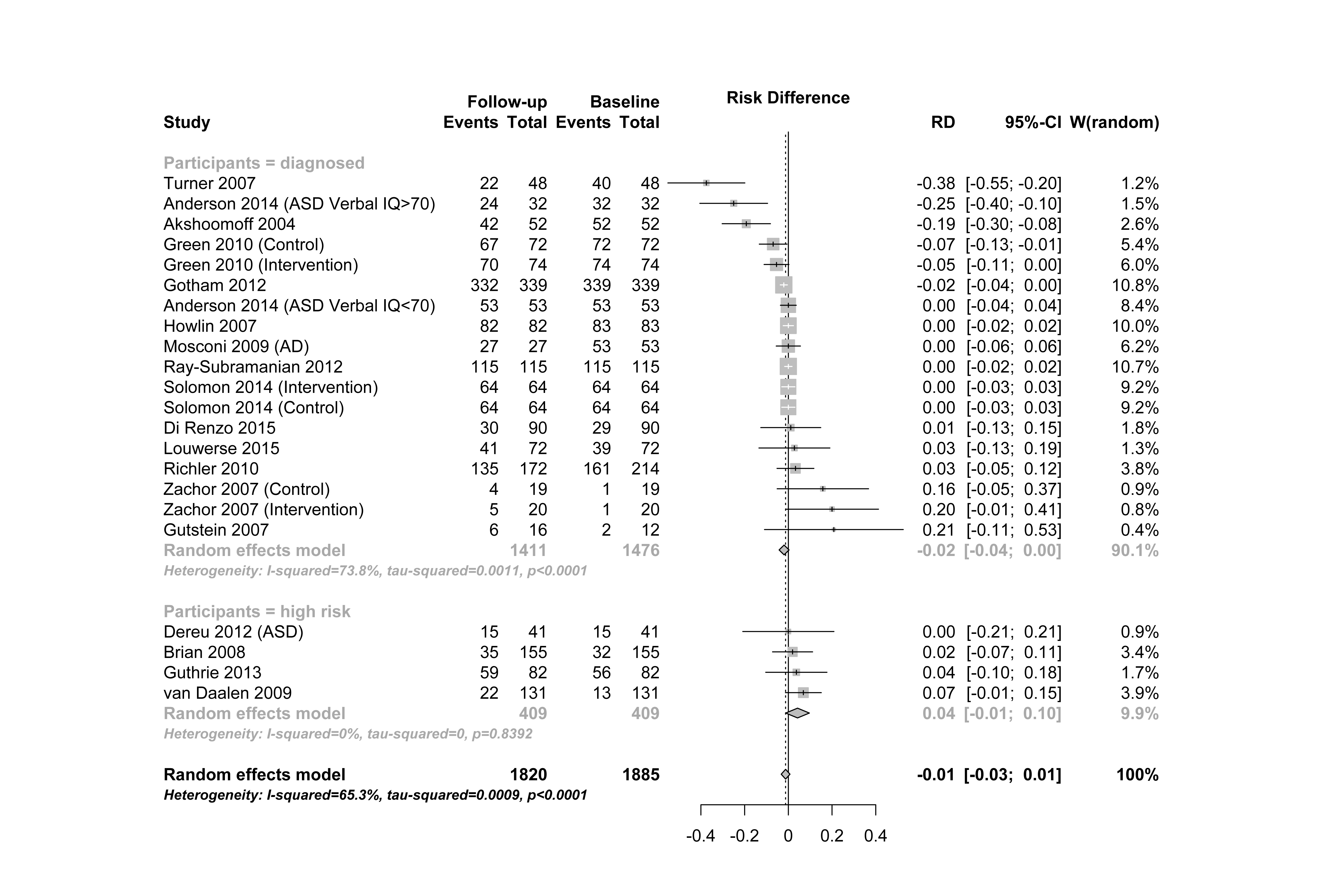

Supplement: S2 Fig — (TIF) [file pone.0183160.s005.tif]
